# Biomarkers of Chlorpyrifos Exposure and Effect in Egyptian Cotton Field Workers

**DOI:** 10.1289/ehp.1002873

**Published:** 2011-01-11

**Authors:** Fayssal M. Farahat, Corie A. Ellison, Matthew R. Bonner, Barbara P. McGarrigle, Alice L. Crane, Richard A. Fenske, Michael R. Lasarev, Diane S. Rohlman, W. Kent Anger, Pamela J. Lein, James R. Olson

**Affiliations:** 1 Department of Community Medicine and Public Health, Faculty of Medicine, Menoufia University, Shebin El-Kom, Egypt; 2 Department of Pharmacology and Toxicology and; 3 Department of Social and Preventive Medicine, State University of New York at Buffalo, Buffalo, New York, USA; 4 Department of Environmental and Occupational Health Sciences, University of Washington, Seattle, Washington, USA; 5 Center for Research on Occupational and Environmental Toxicology, Oregon Health and Science University, Portland, Oregon, USA; 6 Department of Molecular Biosciences, University of California-Davis School of Veterinary Medicine, Davis, California, USA

**Keywords:** acetylcholinesterase, butyrylcholinesterase, chlorpyrifos, cholinesterase inhibition, occupational exposure, urinary TCPy

## Abstract

**Background:**

Chlorpyrifos (CPF), a widely used organophosphorus pesticide (OP), is metabolized to CPF-oxon, a potent cholinesterase (ChE) inhibitor, and trichloro-2-pyridinol (TCPy). Urinary TCPy is often used as a biomarker for CPF exposure, whereas blood ChE activity is considered an indicator of CPF toxicity. However, whether these biomarkers are dose related has not been studied extensively in populations with repeated daily OP exposures.

**Objective:**

We sought to determine the relationship between blood ChE and urinary TCPy during repeated occupational exposures to CPF.

**Methods:**

Daily urine samples and weekly blood samples were collected from pesticide workers (*n* = 38) in Menoufia Governorate, Egypt, before, during, and after 9–17 consecutive days of CPF application to cotton fields. We compared blood butyrylcholinesterase (BuChE) and acetylcholinesterase (AChE) activities with the respective urinary TCPy concentrations in each worker.

**Results:**

Average TCPy levels during the middle of a 1- to 2-week CPF application period were significantly higher in pesticide applicators (6,437 μg/g creatinine) than in technicians (184 μg/g) and engineers (157 μg/g), both of whom are involved in supervising the application process. We observed a statistically significant inverse correlation between urinary TCPy and blood BuChE and AChE activities. The no-effect level (or inflection point) of the exposure–effect relationships has an average urinary TCPy level of 114 μg/g creatinine for BuChE and 3,161 μg/g creatinine for AChE.

**Conclusions:**

Our findings demonstrate a dose–effect relationship between urinary TCPy and both plasma BuChE and red blood cell AChE in humans exposed occupationally to CPF. These findings will contribute to future risk assessment efforts for CPF exposure.

Chlorpyrifos (CPF) belongs to the class of organophosphorus pesticides (OPs), which are the most commonly used pesticides worldwide. CPF produces neurotoxic effects via inhibition of β-esterases, including butyrylcholinesterase (BuChE), acetylcholinesterase (AChE), and carboxylesterase (reviewed by Costa 2006). The oxon metabolite of CPF mediates the inhibition of these β-esterases (Amitai et al. 1998). The inhibition of AChE is accepted as a primary mechanism by which OPs cause neurotoxicity. AChE inhibition increases acetylcholine in both central and peripheral cholinergic synapses, resulting initially in overstimulation of nicotinic and muscarinic receptors followed by receptor down-regulation. Acute cholinergic toxicity (OP poisoning) is generally thought to be mediated by overstimulation of receptors secondary to AChE inhibition, whereas it has been hypothesized that chronic OP neurotoxicity is due to receptor down-regulation (Costa 2006; Mileson et al. 1998). Although brain AChE cannot be practically monitored in humans exposed to CPF, red blood cell (RBC) AChE and plasma BuChE activities are used as biomarkers of effect both in the clinical setting (Thiermann et al. 2007) and the occupational setting (Quandt et al. 2010; Wilson et al. 2009). Plasma BuChE is more sensitive to OP exposure than RBC AChE (Amitai et al. 1998); therefore, BuChE is commonly used to monitor occupational exposure (Khan et al. 2008) and the response of acutely intoxicated individuals to oxime therapy (Aurbek et al. 2009; Wilson et al. 1997).

Measuring cholinesterase (ChE) inhibition is a useful biomarker of OP exposure and effect, but it is not specific to CPF. 3,5,6-Trichloro-2-pyridinol (TCPy), a metabolite excreted in the urine, can be used as a specific biomarker of exposure of methyl and ethyl CPF (Barr et al. 2005). Analysis of urinary TCPy is useful in the occupational setting, as urine is one of the most common samples used for biological monitoring of OP exposure because of its abundance and ease of collection (Albers et al. 2004; Arcury et al. 2010; Barr et al. 1999; Hines and Deddens 2001; Steenland et al. 2000). TCPy concentration in the urine has also been used to assess CPF exposure in epidemiological studies of the general population (Whyatt et al. 2009).

Although substantial data support the validity of urinary TCPy and blood ChE as biomarkers of exposure and effect after acute exposure to high concentrations of CPF, considerably less is known about their ability to predict adverse effects in populations exposed repeatedly to a range of CPF levels. Critical questions for which there are currently little directly relevant data include the relationship of blood ChE and urinary TCPy to exposure level and to each other after repeated occupational exposures. To address these questions, we quantified urinary TCPy levels and blood BuChE and AChE in a group of pesticide application workers in Menoufia Governorate, Egypt. Previous studies in Egyptian agricultural workers have shown that these workers have significant exposures to CPF (Farahat et al. 2010) and exhibit extensive neurobehavioral deficits compared with a control population (Abdel Rasoul et al. 2008; Farahat et al. 2003).

## Methods

### Study setting, population, and pesticide application

**Figure 1:**
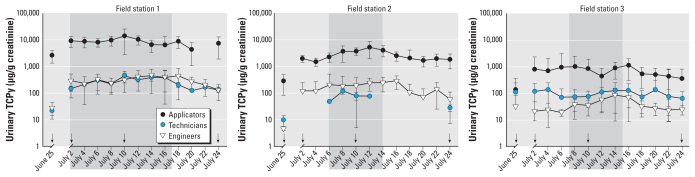
Urinary concentrations of TCPy, a CPF-specific metabolite, in applicators, technicians, and engineers from field stations 1, 2, and 3 during the summer of 2008. Shaded areas represent the period of CPF application; arrows indicate days blood was collected. Data are presented as mean ± SD (*n* = 2–5 workers for each job category in a given field station.

This study follows up on initial observations of Egyptian cotton-field workers (Farahat et al. 2010). In brief, the present study took place in Menoufia, one of 29 governorates in Egypt, which is situated in the Nile River Delta north of Cairo. The Egyptian national government purchases and sells the nation’s entire production of cotton. Consequently, on an annual basis, the Ministry of Agriculture directs the use of pesticides and application procedures in cotton fields throughout Egypt. In July 2008, CPF was applied daily to cotton plants on different fields for 9–17 consecutive days, depending on the area of planted cotton for which a ministry field station was responsible (Figure 1). A field station is located close to the cotton fields and serves as the meeting place for workers and supervisors and as the storage area for pesticides and application equipment. Each field station consists of a team of Ministry of Agriculture employees belonging to one of three job categories: *a*) applicators, who apply pesticides with backpack sprayers; *b*) technicians, who walk each row with the applicator to direct the path of the applicator and point out any heavy infestations in the field; and *c*) engineers, who periodically walk the fields but more often direct the application process from the edge of the field. In our study, three field stations employing a total of 14 applicators, 12 technicians, and 12 engineers were monitored for potential exposure to CPF during the first 24 days of July 2008. This time frame included the first cycle of CPF application in the year and the week following the end of this application cycle (Figure 1).

Each prospective agricultural worker was asked to complete a brief self-administered screening questionnaire to assess his eligibility for inclusion into the study. Prospective participants who were determined to be eligible gave written informed consent prior to enrollment into the study. All enrolled participants were asked to complete a self-administered questionnaire on demographics, education, occupational histories (agricultural and nonagricultural), use of personal protective equipment, and medical history including symptoms of OP toxicity. The demographics, education, occupational histories, and use of personal protective equipment for applicators, technicians, and engineers in each of the three field stations are summarized in Table 1 and Supplemental Material, Table 1 (doi:10.1289/ehp.1002873). All protocols and questionnaires were approved by the institutional review boards of Oregon Health and Science University and Menoufia University.

### Selection of participants

Workers from field stations with > 12 workers and having at least 1 worker in each job category (applicator, technician, or engineer) were eligible to participate. In addition, workers were eligible if they were between 15 and 55 years of age and had been employed in the cotton fields during the previous three seasons. These latter two criteria were included to reduce loss to follow-up. Because certain disease states can adversely influence the metabolism and excretion of TCPy, all workers were questioned about prior diagnosis of diabetes mellitus and liver or kidney disease by a physician during the recruitment process. However, no exclusions for medical conditions were necessary. All of those recruited to participate in the study consented to urine and blood collection except for 4 workers, who were excluded from the study.

### Analytical methods

#### Urine collection and TCPy analysis

During 2008, spot urine samples were collected daily at the beginning and end of the work shift. Samples were placed on wet ice in a cooler and transported to Menoufia University (Shebin El-Kom, Egypt), where they were stored at −20°C until being shipped to the State University of New York at Buffalo (Buffalo, NY, USA) on dry ice for analysis. Urine samples were analyzed for TCPy, the primary metabolite of CPF, by negative-ion chemical ionization gas chromatography–mass spectrometry, using ^13^C-^15^N-3,5,6-TCPy as an internal standard, as described previously (Farahat et al. 2010). Creatinine concentrations were measured using the Jaffe reaction (Fabiny and Ertingshausen 1971); urine TCPy concentrations are expressed as micrograms TCPy per gram creatinine. The within-run imprecision of this assay is very low, as shown by a < 2% coefficient of variation and an intraclass correlation coefficient of 0.997.

#### Blood collection and analysis of BuChE and AChE activity

To establish the baseline ChE activity for each worker, a single preexposure blood sample was collected on 25 June 2008, prior to the start of the official government-regulated CPF application season (Figure 1). Additional blood samples were collected during CPF application, and a post-CPF exposure blood sample was collected when CPF spraying had ended in all three field stations. Blood samples were collected by venipuncture into 10-mL lavender top (EDTA) Vacutainer tubes and immediately placed on wet ice and transported to Menoufia University, where they were analyzed in triplicate for AChE and BuChE activity using an EQM Test-Mate kit (EQM Research Inc., Cincinnati, OH, USA). The EQM Test-Mate kit, based on the original Ellman method (Ellman et al. 1961), is a portable photometric analyzer developed for the determination of ChE activity in whole blood as a basis for monitoring pesticide exposure (McConnell et al. 1992). The intraclass correlation coefficient for BuChE with this method is 0.987 and for AChE is 0.898, indicating that there is very little variation among replicates relative to total variation, which is desirable for a biological assay.

### Statistical analysis

#### Questionnaire data

The means for select characteristics between job categories (applicators, technicians, and engineers) were compared using one-way analysis of variance (ANOVA) with Tukey’s post hoc analysis.

#### Plasma BuChE relationship to urinary TCPy

Plots of plasma BuChE activity against log-transformed TCPy concentration suggested that the SD of BuChE was proportional to its mean response. Consequently, we used the gamma distribution to model these data, with the mean of the distribution following a generalized Michaelis–Menten function. Parameters for the model were fit by maximum likelihood. We estimated the EC_50_ of TCPy (urinary TCPy concentration associated with a 50% decrease in plasma BuChE activity) from the model, as well as the inflection point (the TCPy level at which the mean BuChE experienced the steepest rate of decrease).

#### The relationship between RBC AChE and urinary TCPy

Urinary TCPy concentration and RBC AChE activity were log-transformed prior to analysis and separated by date into four cross-sectional units. A single piecewise linear model relating levels of urinary TCPy and RBC AChE was fit to each set using nonlinear least squares (Bacon and Watts 1971). The model simultaneously supplies estimates for the inflection point between the two linear segments and slopes for each linear component. Confidence intervals (CIs) for parameter estimates were constructed (on the log scale) and then back-transformed to the original scale. Upon back-transformation, inferential statements concerning the median response (e.g., AChE) and additive changes on the log scale become multiplicative effects on the original scale (e.g., percent change per 10-fold increase in TCPy) (Aitkin et al. 2005).

#### Estimating daily internal CPF exposure

Assuming steady-state conditions, it is possible to estimate the daily absorbed dose of CPF (micrograms per kilogram per day) associated with a given urinary TCPy concentration (micrograms per gram creatinine):


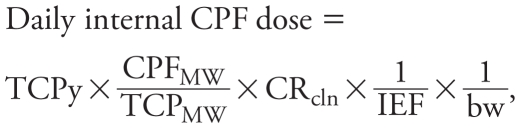


where CPF_MW_ and TCP_MW_ are the molecular weights of CPF (350.6) and TCPy (198.5), respectively; CR_cln_ is the daily creatinine clearance (grams per day); IEF is the incomplete excretion factor for CPF (0.7), which was based on a human oral CPF dosing study (Nolan et al. 1984); and BW is the average body weight of workers in this study (77.9 kg). Individual worker creatinine excretion was estimated using the method of Mage et al. (2004) and averaged to determine creatinine excretion for the population.

## Results

### Study population

Height, weight, body mass index (BMI), and years of smoking were not significantly different across the three job categories of Egyptian cotton-field workers (Table 1). Applicators were significantly younger and had less job experience than technicians and engineers, which was expected because applicators are seasonal workers, often students on summer vacation. Other factors such as education, use of personal protective equipment, and occupational history, were similar among all three job categories [see Supplemental Material, Table 1 (doi:10.1289/ehp.1002873)].

### Urinary TCPy concentrations

Similar to previous findings (Farahat et al. 2010), daily urinary TCPy levels for a given worker were very similar in specimens collected at the beginning and end of a given work day. Urine specimens were more consistently collected at the beginning of the work day; therefore, we used TCPy levels in these samples as the exposure biomarker. Urinary TCPy concentrations, stratified by job category for each field station, are shown in Figure 1. Because 25 June was prior to the start of the government- regulated CPF spray season, urinary TCPy concentrations for that day were used as baseline measurements across all three field stations. All workers, regardless of job category and field station, had detectable levels of TCPy in their urine at baseline. In general, average urinary TCPy concentrations in each job category increased ≥ 300% once CPF application began, compared with respective baseline measurements. Average TCPy levels during the middle of a 1- to 2-week CPF application period (10 July 2008) were significantly higher in applicators (6,437 μg/g creatinine) than in technicians (184 μg/g) and engineers (157 μg/g). Among the three field stations monitored for urinary TCPy excretion, workers at field station 1 had the highest urinary TCPy concentrations. Workers at this field station were responsible for pesticide application to the largest area of cotton production in the district and had more work days and/or longer work days and thus a greater CPF exposure than the other two field stations. Upon cessation of CPF application, urinary TCPy concentrations decreased over time. However, relative to baseline levels, average urinary TCPy concentrations remained elevated 8–10 days after the last day of CPF application.

### Cholinesterase activity

The plasma BuChE and RBC AChE activities measured in blood samples collected before (baseline), during, and after the CPF application cycle are shown in Table 2. Baseline measurements of BuChE activity ranged widely, depending on the field station and job classification of the worker. Once CPF application began, BuChE activity was suppressed relative to baseline measurements in most workers, regardless of field station or job category. We observed a statistically significant decrease in BuChE activity in only a few groups of workers because of small sample sizes when stratifying by job category and field station. In all three field stations, applicators experienced the greatest amount of BuChE inhibition, followed by technicians and engineers (Table 2). Eight to 10 days after the designated CPF application period had ended, BuChE activity remained inhibited relative to baseline measurements across all workers.

Baseline RBC AChE activities were similar for all workers across all three field stations and between job categories, with average values ranging from 23 to 28 U/g hemoglobin. Once CPF spraying began, applicators from field station 1 were the only group to show a statistically significant decrease in AChE activity (Table 2).

### Relationship between urinary TCPy and BuChE activity

**Figure 2:**
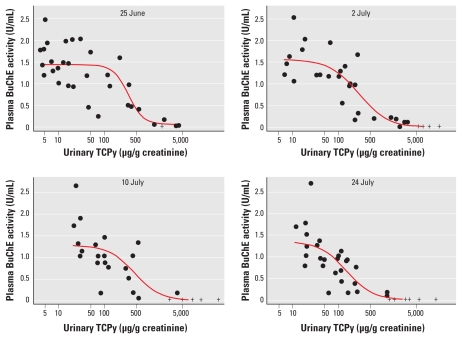
Plasma BuChE activity plotted against urinary TCPy concentration for Egyptian agricultural workers before (25 June), during (2 July and 10 July), and after (24 July) CPF application. Data points represent paired matched values for BuChE activity and urine TCPy content from individual workers. Plus symbols (+) represent values below the level of detection for BuChE (0.02 U/mL), which we estimated to be between 0 and 0.02 U/mL.

To determine whether there was a relationship between urinary TCPy concentration and plasma BuChE activity, we plotted BuChE activities relative to the respective urinary TCPy level for each worker at each of four time points when blood was collected (25 June and 2, 10, and 24 July) (Figure 2). We observed a concentration-dependent inverse relationship between urinary TCPy and plasma BuChE. Several workers had urinary TCPy concentrations > 1,000 μg/g creatinine, all of which were associated with a plasma BuChE activity between 0 and 0.18 U/mL. Regression analysis of the data from 2 July and 10 July identified urinary TCPy concentrations of 67 and 161 μg/g creatinine, respectively, as corresponding to an inflection point, representing a significant decrease in BuChE activity (Table 3).

### Relationship between urinary TCPy and AChE activity

**Figure 3:**
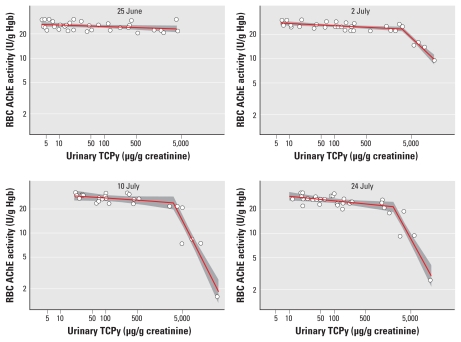
RBC AChE activity plotted against urinary TCPy concentration for Egyptian agricultural workers before (25 June), during (2 July and 10 July), and after (24 July) CPF application. Hgb, hemoglobin. Data points represent paired matched values for AChE activity and urine TCPy content from individual workers. Red lines represent results from the piecewise linear model, and gray shading represents 95% CIs.

Paired match values for AChE activity and urinary TCPy concentration for each worker were plotted at four time points when blood was collected (Figure 3). Urinary TCPy concentrations at baseline (25 June)ranged from 4.1 to 4,080 μg/g creatinine; however, we observed a nonsignificant trend in decreased RBC AChE activity with increased TCPy concentration. With the initiation of CPF application on 2 July, urinary TCPy concentrations increased (from 6.8 to 15,840 μg/g creatinine), and we identified an inflection point representing a significant decrease in AChE activity of 3,148 μg TCPy/g creatinine (Table 4). A similar inflection point for AChE inhibition (3,173 μg/g creatinine) was observed on 10 July. AChE activity was still inhibited up to 10 days after the CPF spray period ended, even though urinary TCPy concentrations were decreasing. The workers in field station 1 had the highest urinary TCPy concentrations and the greatest decrease in AChE activity (Figure 1, Table 2). When the model was controlled for potential confounders, we found no significant impact of age, smoking, or BMI on the relationship between AChE activity and urinary TCPy levels.

### Estimating internal CPF dose

Assuming steady-state conditions, we used Equation 1 to estimate internal CPF dose at a given urinary TCPy concentration. The average (2 July and 10 July) urinary TCPy concentration inflection point for BuChE inhibition was 114 μg/g creatinine (Table 3), which corresponds to an estimated internal CPF dose of 6.5 μg/kg BW/day. The average (2 July and 10 July) urinary TCPy concentration inflection point for AChE inhibition was 3,161 μg/g creatinine (Table 4), which corresponds to an estimated internal CPF dose of 181 μg/kg BW/day.

## Discussion

### TCPy concentrations after daily CPF exposures

In this study of Egyptian cotton field workers, we observed a wide range of urinary TCPy levels compared with previous studies [see Supplemental Material, Table 2 (doi:10.1289/ehp.1002873)]. The mean level of urinary TCPy for field station 1 applicators (8,851 μg/g creatinine) during CPF application was 3.5 times higher than previously reported exposures for Egyptian pesticide application workers (Farahat et al. 2010), > 33 times higher than reported levels in termite control applicators in the United States (Hines and Deddens 2001), approximately 150 times higher than in CPF manufacturers in the United States (Garabrant et al. 2009), > 840 times higher than that reported for farm applicators in the United States (Alexander et al. 2006), and about 4,155 times higher than background levels reported in the United States (Centers for Disease Control and Prevention 2009).

The elevated urinary TCPy levels in some workers prior to CPF application (see data for 25 June) can be explained in part by the workers’ episodic use of pesticides outside of their ministry jobs. The relatively small within-worker differences in daily urinary TCPy levels throughout the study suggest that these workers were approaching a steady-state exposure to CPF. The substantial between-worker variability in TCPy excretion indicates that there was a wide exposure range to CPF, which likely reflects factors such as job category, field station, work habits, and environmental conditions (e.g., wind direction), as well as interindividual differences in CPF metabolism (Foxenberg et al. 2007), which could influence urinary excretion of TCPy.

In general, urinary TCPy concentrations remained elevated from baseline for ≥ 7 days after CPF spraying had ceased (Figure 1). This was an unexpected finding, based on the reported 26.9-hr half-life for urinary elimination of TCPy (Nolan et al. 1984). There are several possible explanations: *a*) The workers do not regularly wash their CPF-contaminated clothing, thus providing a route for CPF exposure in the absence of spraying CPF; *b*) workers from all three job categories use CPF outside their ministry jobs; *c*) CPF residue may persist on the cotton plants even after CPF spraying has ended, thus allowing for foliage-to-skin transfer of CPF whenever the workers walk through cotton fields; and *d*) the pharmacokinetics of CPF metabolism and excretion may be different after a sustained exposure to CPF, as seen in our worker population, compared with short-term exposures, which have been used previously to determine the half life of CPF (Griffin et al. 1999; Nolan et al. 1984).

### Decreased ChE after daily CPF exposure

Baseline determination of BuChE activity showed that there was already inhibition in the BuChE enzyme activity of some of the workers. This agrees with baseline urinary TCPy concentrations, adding to the evidence that some workers were exposed to CPF prior to the actual start of the CPF application cycle in the cotton fields. Worker BuChE activity was further suppressed during the CPF application cycle and remained depressed 8–10 days after the ministry’s CPF application cycle had ended. At the beginning of the study, baseline AChE activity was comparable across all workers and within the normal interindividual variability range of 10–15% (Kamanyire and Karalliedde 2004). Once CPF application began, AChE activity was depressed in field station 1 applicators, whose average AChE activity on 2 July, 10 July, and 24 July was inhibited by 43, 73, and 70%, respectively.

It is interesting to note that on 10 July five workers had RBC AChE activities that were depressed by > 60% compared with baseline measurements; however, they continued to work their full shifts in the cotton fields. When two of these workers with the greatest depression were questioned about their health, neither reported problematic symptoms. How these workers could experience such large depressions in RBC AChE activity but show no outward sign of toxicity is unclear, but lack of symptoms suggests a mechanism of physiological adaptation. Kwong (2002) suggested that repeated doses to an OP pesticide can gradually decrease AChE activity to very low levels in the absence of overt toxic symptoms; thus, very low levels of ChE activity do not always correlate with overt toxicity.

### Dose–effect relationship between TCPy and blood ChE measures

Few studies have attempted to identify a dose–effect relationship between urinary TCPy concentration and blood ChE activity in humans after occupational exposure to CPF. Recently, Garabrant et al. (2009) examined this relationship in CPF manufacturing workers and identified a no-effect level of 110 μg TCPy/g creatinine for BuChE inhibition but not for AChE inhibition. Our data from samples collected during the CPF application cycle (2 July and 10 July) show a similar average inflection point of urinary TCPy (114 μg/g creatinine) associated with BuChE inhibition (Table 3). At much higher urinary TCPy levels, a consistent dose–effect relationship for inhibition of AChE was demonstrated. During CPF application (2 July and 10 July), an average inflection point of 3,161 μg/g creatinine was observed (Table 4). These data from humans exposed occupationally to CPF demonstrate a dose-related correlation between urinary TCPy and not only plasma BuChE, which is consistent with the findings of Garabrant et al. (2009), but also RBC AChE. RBC AChE is considered to provide a more accurate reflection of brain AChE than plasma BuChE (Eaton et al. 2008); therefore, the finding of a dose-related correlation between urinary TCPy and RBC AChE is significant because it identifies a level of urinary TCPy that potentially reflects adverse neural effects in humans after occupational exposures to CPF. Our ongoing studies of this occupational cohort will directly assess the relationship between neurobehavioral function and the well defined dose–effect relationships between urinary TCPy and both plasma BuChE and RBC AChE.

We found that the inflection points for BuChE and AChE inhibition were associated with lower urinary TCPy levels a week or more after the end of the CPF application cycle (24 July) (Tables 3 and 4). This observation highlights the importance of considering the relationship between the duration and timing of exposure to the time samples are collected for biomarker measurements. In this case, it is likely that urinary TCPy levels were decreasing postapplication at a greater rate than ChE activity was recovering.

### Comparison with previously established worker protection guidelines

The short-term (i.e., 1–30 days) human no observed adverse effect level (NOAEL) for a dermal CPF exposure was set by the U.S. Environmental Protection Agency (EPA) at 5 mg/kg/day based on a rat study in which plasma BuChE and RBC AChE were inhibited 45% and 16%, respectively (U.S. EPA 2006). To calculate a NOAEL for CPF suitable for comparison with biologically based dose estimates, the short-term dermal NOAEL of 5 mg/kg/day was adjusted by a 0.03 dermal absorption factor, resulting in a NOAEL at an internal dose of 150 μg/kg/day (U.S. EPA 2006). The internal CPF dose for this Egyptian population associated with the average AChE inflection point (urinary TCPy level of 3,161 μg/g creatinine) was 181 μg/kg/day which is similar to the rat-based NOAEL set by the U.S. EPA.

With respect to the short-term human NOAEL (internal CPF dose of 150 μg/kg/day), the U.S. EPA has a 100-fold margin of exposure (MOE) target for occupational exposure due to interspecies uncertainties and variability (U.S. EPA 2006). Thus, the U.S. EPA target MOE dose of ≤ 1.5 μg/kg/day is a target level used in occupational risk assessment. Based on the results of the present study, the BuChE inflection point (urinary TCPy concentration of 114 μg/g creatinine) would be equivalent to an internal CPF dose of 6.5 μg/kg/day. This value is approximately 4 times higher than the U.S. EPA MOE target (1.5 μg/kg/day). This suggests that the U.S. EPA MOE target is protective for both BuChE and AChE inhibition.

## Conclusions

Our findings in the present study are the first to demonstrate a dose–effect relationship between urinary TCPy concentrations and the inhibition of both plasma BuChE and RBC AChE activity in humans occupationally exposed to CPF. This dose–effect relationship can be further used to guide future risk assessment efforts for CPF exposure. These findings will also be important in educating Egyptian pesticide application workers about the extent of CPF exposure in order to encourage the development and implementation of work practices and personal protective equipment to reduce CPF exposures.

## Figures and Tables

**Table 1:** Occupational survey of the study population during the summer of 2008.

Characteristic	Applicators (*n* = 14)	Technicians (*n* = 12)	Engineers (*n* = 12)	ANOVA *p*-Value
Age (years)	25.1 ± 11.4#	48.8 ± 3.8	46.3 ± 3.2	< 0.0001
Height (cm)	169.5 ± 6.5	169.8 ± 5.0	172.8 ± 2.8	0.227
Weight (kg)	73.5 ± 14.6	79.5 ± 9.7	81.6 ± 12.6	0.247
Body mass index (kg/m^2^)	25.4 ± 3.7	27.5 ± 2.4	27.3 ± 4.1	0.263
Smoking (pack-years)	1.4 ± 3.2	3.2 ± 3.9	4.6 ± 3.7	0.074

Values shown are mean ± SD.

# *p* < 0.0001 compared with the two other job categories, determined by one-way ANOVA with Tukey’s post hoc analysis.

**Table 2:** Average BuChE and AChE activity from Egyptian agricultural workers before (25 June), during (2 July and 10 July), and after (24 July) CPF application (mean ± SD).

	Field station 1			Field station 2			Field station 3		
Measure, date	Applicators (*n =* 5)	Technicians (*n =* 5)	Engineers (*n =* 4)	Applicators (*n =* 4)	Technicians (*n =* 2)	Engineers (*n =* 3)	Applicators (*n =* 5)	Technicians (*n =* 5)	Engineers (*n =* 5)
Plasma BuChE (U/mL)									
25 June	0.07 ± 0.06	1.55 ± 0.29	0.99 ± 0.40	1.18 ± 0.61	1.49 ± 0.00	1.55 ± 0.31	1.45 ± 0.61	0.83 ± 0.46	1.79 ± 0.53
2 July	0.00 ± 0.04	0.74 ± 0.67	0.67 ± 0.48	0.13 ± 0.08*	1.38 ± 0.04	1.61 ± 0.38	1.22 ± 0.76	0.85 ± 0.50	1.66 ± 0.55
10 July	0.00 ± 0.00	0.62 ± 0.20*	0.40 ± 0.37*	0.05 ± 0.08*	1.15 ± 0.00	1.13 ± 0.30	1.00 ± 0.54	0.74 ± 0.56	1.67 ± 0.68
24 July	0.00 ± 0.00*	0.79 ± 0.23	0.53 ± 0.33*	0.02 ± 0.04*	1.24 ± 0.00	1.18 ± 0.18	0.59 ± 0.38*	0.85 ± 0.62	1.56 ± 0.76

RBC AChE (U/g Hgb)									
25 June	23.7 ± 4.5	25.5 ± 3.9	24.4 ± 2.7	23.2 ± 2.0	27.1 ± 2.6	25.7 ± 4.1	27.7 ± 2.3	24.7 ± 1.5	27.2 ± 3.6
2 July	13.6 ± 2.8*	27.1 ± 3.7	25.9 ± 2.7*	24.5 ± 2.1	24.0 ± 1.6	25.4 ± 3.6	26.5 ± 2.2	24.9 ± 1.4	27.8 ± 2.3
10 July	6.5 ± 2.8*	27.3 ± 6.1	26.5 ± 3.4	22.0 ± 1.3	26.0 ± 3.3	26.6 ± 4.3*	28.7 ± 2.8	25.9 ± 2.2	29.4 ± 1.3
24 July	7.1 ± 3.9	25.4 ± 2.9	27.0 ± 2.3*	20.8 ± 2.6	26.7 ± 0.0	24.7 ± 3.5	26.4 ± 3.2	25.8 ± 2.1	29.8 ± 2.5

* *p* < 0.05. compared with the baseline (25 June) activities for each group by job category and field station, determined by paired *t*-test.

**Table 3:** Estimated parameters relating urinary TCPy concentration (μg/g creatinine) to plasma BuChE activity before (25 June), during (2 July and 10 July), and after (24 July) CPF application.

Date	EC_50_ (95% CI)	Inflection point
25 June (*n* = 33)	333 (209–530)	248
2 July (*n* = 31)	274 (118–641)	67
10 July (*n* = 27)	448 (118–1,700)	161
24 July (*n* = 33)	149 (60.8–365)	49.5

**Table 4:** Estimated parameters relating urinary TCPy concentrations to blood AChE activity before (25 June), during (2 July and 10 July), and after (24 July) CPF application.

Date	Percent change in blood AChE activity (95% CI)^a^		Transition [inflection point (95% CI)]^b^
	Initial segment	Second segment	
25 June (*n* = 33)	−4.2 (−8.5 to 0.30)		
2 July (*n* = 31)	−6.2 (−10 to −1.7)*	−71 (−80 to −59)**	3,148 (2,263 to 4,377)
10 July (*n* = 27)	−8.8 (−23 to 8.7)	−92 (−96 to −86)**	3,173 (2,210 to 4,556)
24 July (*n* = 33)	−12 (−22 to −1.9)*	−91 (−95 to −82)**	2,044 (1,394 to 2,997)

a Percent change in AChE activity (U/g hemoglobin) for every 10-fold increase in urinary TCPy concentrations (μg/g creatinine).

b μg TCPy/g creatinine.

* *p* ≤ 0.05, and

** *p* ≤ 0.001 for the slope of each line segment that is significantly different from horizontal [zero slope (i.e., the line segment slope, including the 95% CI for AChE activity, does not include zero)].
